# Bioconversion of spent coffee grounds to prebiotic mannooligosaccharides – an example of biocatalysis in biorefinery

**DOI:** 10.1039/d2ra07605e

**Published:** 2023-01-26

**Authors:** Mihle Magengelele, Samkelo Malgas, Brett I. Pletschke

**Affiliations:** a Enzyme Science Programme (ESP), Department of Biochemistry and Microbiology, Rhodes University Makhanda (Grahamstown) 6140 Eastern Cape South Africa b.pletschke@ru.ac.za; b Department of Biochemistry, Genetics and Microbiology, University of Pretoria Hatfield 0002 Gauteng South Africa

## Abstract

Spent coffee ground (SCG), an agro-industrial waste, have a high content of polysaccharides such as mannan, making it ideal for utilisation for the production of nutraceutical oligosaccharides. Recently, there has been growing interest in the production of mannooligosaccharides (MOS) for health promotion in humans and animals. MOS are reported to exhibit various bioactive properties, including prebiotic and antioxidant activity. In this study, SCG was Vivinal pretreated using NaOH, characterized and hydrolysed using a *Bacillus* sp. derived *endo*-β-1,4-mannanase, Man26A, for MOS production. Structural analyses using Fourier-transform infrared spectroscopy (FT-IR) and thermogravimetric analysis (TGA) were conducted to assess the efficacy of the pretreatment. Lignin removal by the pretreatment from SCG was clearly shown by TGA. FT-IR, on the other hand, showed the presence of α-linked d-galactopyranoside (812 cm^−1^) and β-linked d-mannopyranoside residues (817 cm^−1^) in both SCG samples, signifying the presence of mannan. Hydrolysis of pretreated SCG by Man26A produced mannobiose and mannotriose as the main MOS products. The effect of simulated gastric conditions on the MOS was investigated and showed this product to be suitable for oral administration. Finally, the prebiotic effect of the MOS on the growth of selected beneficial bacteria was investigated *in vitro*; showing that it enhanced *Lactobacillus bulgaricus*, *Bacillus subtilis* and *Streptococcus thermophilus* growth. These findings suggest that SCG is a viable source for the production of MOS which can be orally administered as prebiotics for effecting luxuriant growth of probiotic bacteria in the host's digestive tract, leading to a good health status.

## Abstract 2

Spent coffee grounds can be bioconverted *via* Man26A to prebiotic mannooligosaccharides; this is an example of biocatalysis in the biorefinery.

## Graphical abstract

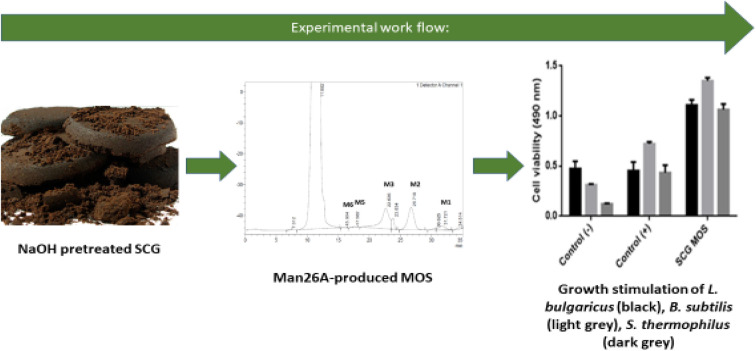

## 1 Introduction

Coffee is currently the second most popular beverage globally, after water.^1,2^ During the preparation of coffee, coffee beans are first roasted and then ground. After this, the coffee is extracted *via* a thermal water extraction technique at 100 and 180 °C.^3^ The resulting water-soluble solids are evaporated and dried either by spray drying or freeze-drying to obtain instant coffee.^3^ The waste, which is the insoluble material, referred to as spent coffee ground (SCG), is then discarded.^3^ Approximately 6 megatons of coffee beans are produced annually across the globe, with a ton of beans usually resulting in 650 kilograms of SCG waste during coffee processing.^4^

Among the SCG components, galactomannan is the major polysaccharide in SCG that accounts for 20–30% dry matter, which is responsible for the high viscosity of the coffee extract.^5,6^ Galactomannan is made up of linear chains of β-1,4-linked mannopyranoside residues substituted by galactopyranoside units at C-6 *via* α-1,6-linkages.^5^ Galactomannans are used in foods as thickening and binding agents, stabilizers in products such as ice cream, dietary fibre and as precursors for the production of nutraceutical mannooligosaccharides (MOS).^1,7^

It is reported that prebiotic oligosaccharides can be found naturally in foods or, alternatively, they can be produced by enzymatic or chemical synthesis from disaccharides or other substrates, as well as by hydrolysis of polysaccharides.^8^ Hydrolysis of polysaccharides is normally the most reliable choice for oligosaccharide production on a large scale, due to its reproducibility and high yield. *Endo*-β-1,4-mannanases (Enzyme Commission number 3.2.1.78) randomly hydrolyse internal β-1,4-glycosidic bonds in the β-mannan backbone, resulting in the production of MOS.^1,4,9^*Endo*-β-1,4-mannanases are currently allotted in glycoside hydrolase (GH) families 5, 26, 45, 113 and 134 in the Carbohydrate-Active enZyme (CAZy) database (https://www.cazy.org) based on sequence similarities.

Before enzymatic hydrolysis, lignocellulosic biomass needs to be pretreated to remove lignin which hampers hydrolysis.^10^ The availability of lignin in SCG prevents CAZymes from accessing and degrading the carbohydrate fraction for the production of free sugars.^11^ This, therefore, necessitates pretreatment of agro-industrial wastes to lower their recalcitrance against enzymatic digestion. Among pretreatment procedures, alkaline pretreatment has low severity and results in more reducing sugars compared to other methods, such as acid pretreatment.^11,12^ Sodium hydroxide (NaOH) pretreatment is reported to result in pores on the surface of SCG, allowing enzymes access for its effective degradation.^11^

MOS have also been reported to have anti-oxidant activity, anti-inflammatory and anti-cancer effects.^9,13^ Chiyanzu *et al.*^14^ reported that MOS can be produced enzymatically using a mannanase and cellulase cocktail from steam pretreated SCG. Nguyen *et al.*^2^ also demonstrated that enzymatic hydrolysis of delignified and defatted SCG resulted in mannobiose (M2) and mannohexaose (M6) as the predominant sugars produced. Wongsiridetchai *et al.*^11,15^ demonstrated that enzymatic hydrolysis of pretreated SCG using a *Bacillus subtilis* GA2 (1) derived mannanase resulted in the production of M2 and mannotriose (M3) as predominant sugars. The MOS produced from both studies enhanced the growth of beneficial bacteria, such as *Lactobacillus acidophilus*, *L. casei* and *L. plantarum*.

This paper investigated the prebiotic properties of MOS produced enzymatically from NaOH pretreated SCG using an *endo*-1,4-β-mannanase from a *Bacillus* sp., including the ability of the MOS to tolerate *in vitro* gastrointestinal conditions and thermal tolerance, which has not been assessed to date. This study showed that the SCG derived MOS indeed exhibited prebiotic effects, including probiotic growth enhancement, their biofilm formation and auto-aggregation improvement, and short chain fatty acid production, which are required by the probiotic for competing with pathogens in the gastrointestinal tract.

## 2 Results and discussion

### 2.1. Pretreatment of SCG

SCG was pretreated using NaOH to make it easier for the *endo*-1,4-β-mannanase to hydrolyse its mannan, by creating pores and increasing the surface area of the biomass.^11^ There was a significant weight reduction of SCG after pretreatment, from 50 g to 30.3 g; while the untreated SCG mass only marginally decreased from 50 g to 47.14 g during incubation in hot water. This mass decrease in the pretreated SCG (39.41% (w/w)) is likely due to the removal of lipids and polyphenolics as reported in a previous study on the saponification of SCG.^11^ Tsai *et al.*^16^ reported that SCG contains 39.4% total lignin, while Ballesteros *et al.*^17^ reported that 23.90% lignin is contained in SCG. We suspect that the alkaline pretreatment was responsible for removal of the lignin from the SCG in the current study. On the other hand, McNutt & He^18^ reported that polysaccharides make up 50% of dry mass of SCG. Furthermore, the SCG polysaccharides are fractionated to 12.40% cellulose and 39.10% hemicellulose, respectively.^17^ The slight mass decrease in untreated SCG (5.72% (w/w)) is most likely due to loss of extractives.

### 2.2. Structural analysis of SCG

#### 2.2.1. FT-IR analysis

**Fig. 1:**
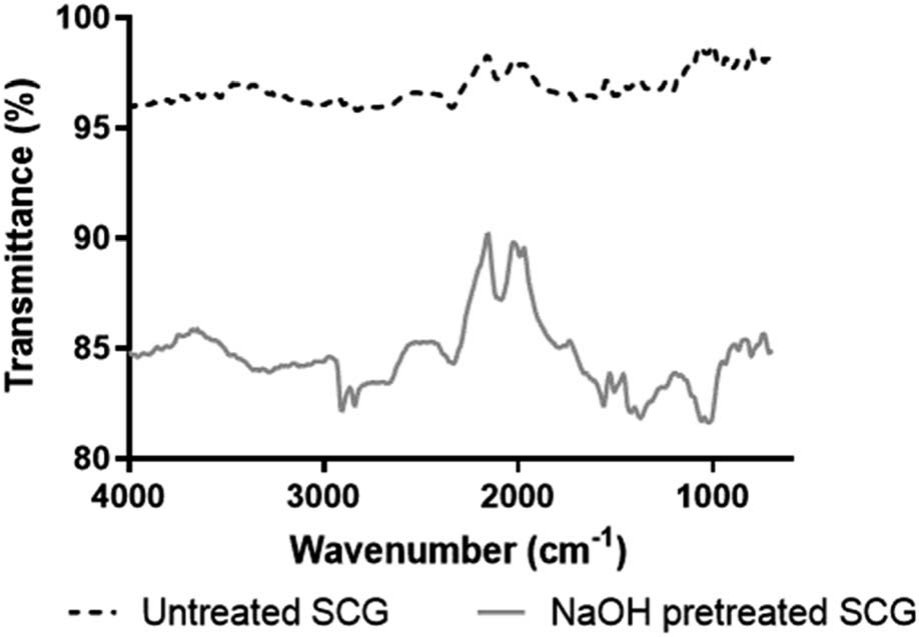
FT-IR spectra of untreated and NaOH pretreated SCG.

FT-IR analysis was conducted to analyse the functional group differences in the untreated and NaOH pretreated SCG (Fig. 1). As shown in Fig. 1, the broad peak at 3364 cm^−1^ is attributed to the O–H vibration in pretreated SCG, which is usually found in all carbohydrate containing samples and has also been reported at 3277 and 3276 cm^−1^ in coffee waste before oil extraction and after extraction, respectively.^19^ Pandey *et al.*^20^ associated the band at 3426 cm^−1^ with primary (–CH–OH) and secondary (–CH_2_–OH) stretching of hydroxyl vibrations. The peaks at 2914 and 2849 cm^−1^ are associated with CH and CH_2_ stretching vibrations, and this peak is similar to the peak observed by Pandey *et al.*^20^ at 2927 cm^−1^ for locust bean gum. They described the peak observed at 1383 cm^−1^ as the peak formed as a result of deformation of the CH–OH group, and a similar peak was observed at 1416 cm^−1^ in pretreated SCG. There is a peak observed at 1080 cm^−1^ in untreated SCG and a scissor peak at 1071 cm^−1^ in pretreated SCG, these peaks are associated with the C–O (carbon–oxygen stretch) vibration in C–O–H bonds (glycosidic bonds). These bands are associated with the galactomannan in SCG.^17,19,20^ Pandey *et al.*^20^ reported that the peaks at 812 and 817 cm^−1^ show the presence of α-linked d-galactopyranoside units and β-linked d-mannopyranoside units, respectively. The peaks were present in both SCG samples in this study, indicating that galactomannan was not lost during the pretreatment of SCG. The intensity of a peak (1080 cm^−1^), which was attributed to the glycosidic bond (–C–O–H), was more pronounced in pretreated SCG. These findings suggested that hemicellulose was retained and more exposed in pretreated SCG.

#### 2.2.2. TGA analysis

**Fig. 2:**
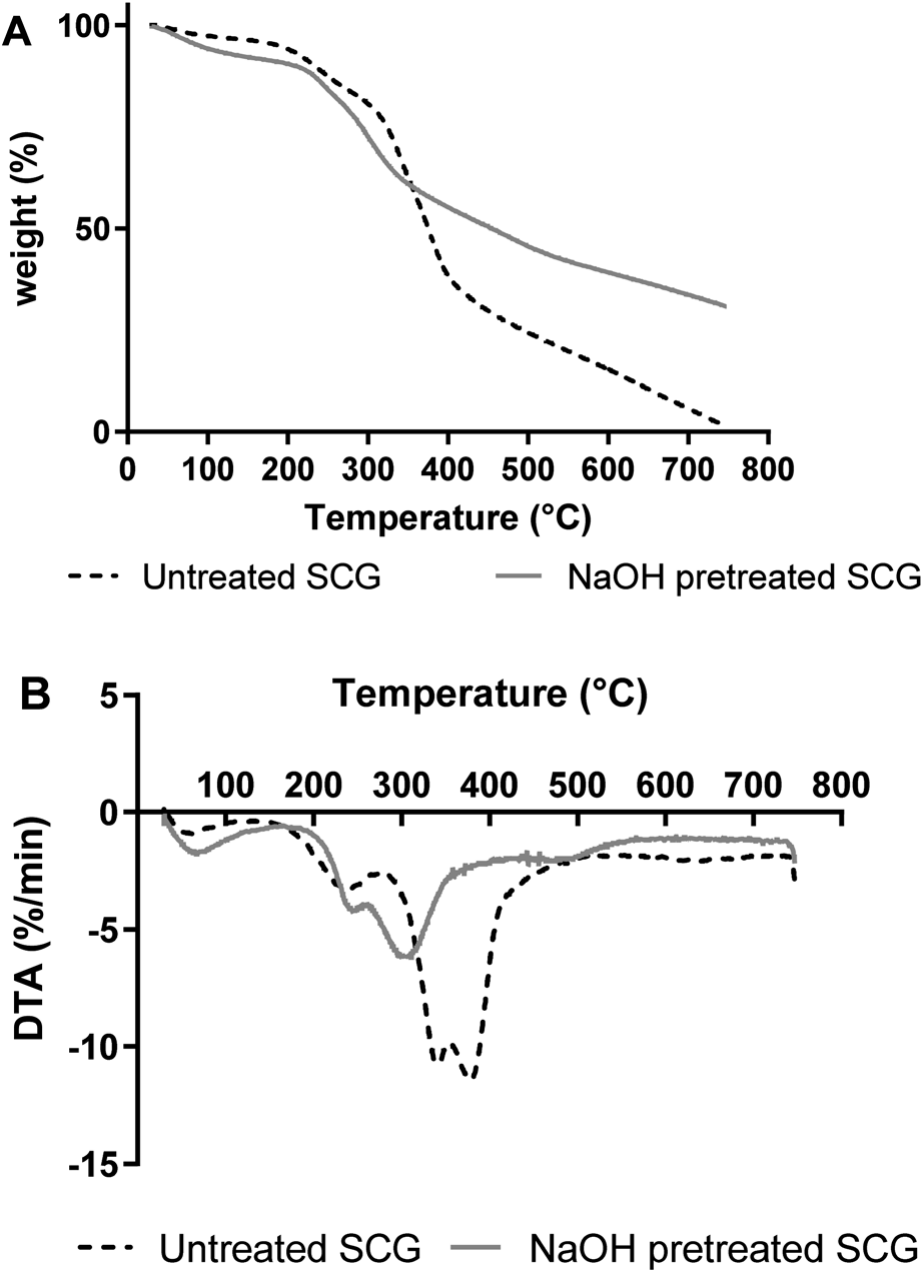
TGA (A) and DTA (B) curves obtained for pretreated and untreated SCG.

TGA analysis was performed on both the pretreated and untreated SCG. Both pretreated and untreated SCG slightly lost weight from approximately 50 °C and this was due to dehydration of the sample. Water and volatile substances are lost at this stage of TGA analysis.^21^ These findings are similar to the findings of Atabani *et al.*^19^ and Ballesteros *et al.*,^17^ who reported that SCG (untreated), SCG before oil extraction and SCG after extraction first lose weight at approximately 60, 60.38 and 77.03 °C, respectively. The biomasses further underwent a massive weight loss between 200 °C and 400 °C. This is the second stage of TGA, where decomposition and depolymerisation of biomass components occur. Ballesteros *et al.*^17^ also showed that SCG undergoes a massive weight loss at approximately 300 °C. Between 200 °C and 400 °C, there are two and three peaks observed in the DTA curves of pretreated and untreated SCG, respectively. There is a hemicellulose peak in both pretreated and untreated SCG at approximately 250 °C, with that of pretreated SCG being slightly increased. DTA also showed a cellulose peak at 300 °C in pretreated SCG, but there was no peak in untreated SCG. DTA then showed a major peak on untreated SCG, between 310 and 400 °C, and this is a lignin peak. The presence of a major lignin peak in the untreated SCG and its absence in the pretreated SCG shows that the removal of lignin *via* NaOH pretreatment was successful. These results are similar to those findings of Carrier *et al.*^21^ regarding DTA analysis of fern components, with decomposition of biomass occurring at 200–300 °C for hemicellulose, followed by decomposition of cellulose at 250–350 °C and lignin at 300–500 °C. Based on the DTA results, pretreated SCG (exothermic peaks around 300 °C) contains less lignin than the untreated SCG (exothermic peaks between 300 °C and 400 °C) (Fig. 2).

### 2.3. Analysis of SCG hydrolysis products by HPLC

**Fig. 3:**
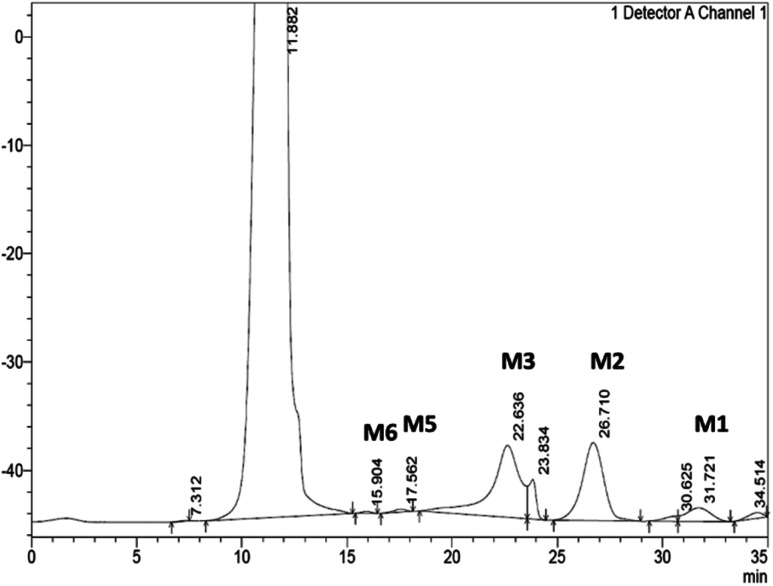
A representative chromatogram displaying the simultaneous detection and separation of MOS released by SCG hydrolysis. The hydrolysis products were separated using a CarboSep CHO 411 column at a flow rate of 0.3 min mL^−1^. Distilled water was used as a mobile phase and using a refractive index (RI) detector. MOS: M1-mannose, M2-mannobiose, M3-mannotriose, M4-mannotetraose (not detected), M5-mannopentaose and M6-mannohexanose.

From the hydrolysis of 40 mg mL^−1^ of SCG by the *Bacillus* sp. derived Man26A over 48 hours, approximately 1.8 mg mL^−1^ of reducing sugars was detected using the DNS assay and 2.47 mg mL^−1^ of MOS could be quantified by HPLC (Table 1). The hydrolysis products of Man26A action against SCG were qualitatively and quantitatively analysed using HPLC. The hydrolysis of this agro-industrial waste using the *Bacillus* sp. Man26A released M2 and M3 as the predominant MOS species (Table 1). This agreed with the findings of Chauhan *et al.*,^22^ who also reported that M2 and M3 were the predominant MOS produced from coffee extract using a *Bacillus nealsonii* PN-11 β-mannanase. Wongsiridetchai *et al.*^11,15^ also reported that SCG hydrolysis, using a *Bacillus* sp. GA2 (1) mannanase, results in M2 and M3 as the predominant products. It is worth mentioning that the MOS yield from SCG hydrolysis by Man26A in this study is an underestimate, as there were two major peaks at 23.8 minutes and 30.6 min, respectively, which were unidentified (Fig. 3). We suspect these two products to be galactosyl substituted MOS, such as galactosyl-mannose and galactosyl-mannobiose. Our previous work has reported on the ability of GH26 *endo*-β-1,4-mannanases to generate galactosyl substituted MOS during the hydrolysis of galactomannans.^9^ The various sizes of oligosaccharides produced from SCG show that the *Bacillus* sp. derived *endo*-β-1,4-mannanase is suitable for MOS production from the inexpensive and abundant SCG.

**Table 1:** HPLC results showing the concentration of MOS (mg mL^−1^) produced as a result of Man26A hydrolysis. Values are represented as means ± standard deviations, *n* = 3. Where “Nd” = not detected and “+” = detected but not quantified

Mannooligosaccharide (MOS) product	Content (mg mL^−1^)
Mannose	0.18 ± 0.03
Mannobiose	1.04 ± 0.03
Mannotriose	1.20 ± 0.04
Mannotetraose	Nd
Mannopentaose	0.03 ± 0.00
Mannohexaose	0.02 ± 0.00
Galactosyl-MOS	+
Total reducing sugars	1.80

### 2.4. Gastrointestinal tolerance of MOS

**Fig. 4:**
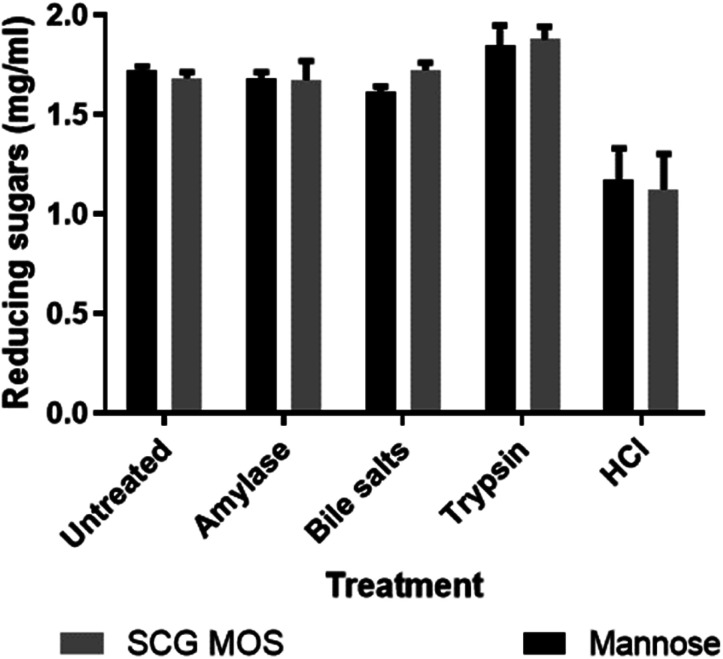
Effect of bile salts, α-amylase, trypsin and hydrochloric acid (pH 1.5) on SCG MOS. Values are represented as means ± standard deviations, *n* = 4. An equal amount mannose (1.7 mg mL^−1^) was treated the same as the MOS to test their recovery post treatment.

Prebiotics must also have several other important characteristics, such as tolerance to acids, bile salts and digestive enzymes that may be present in the gastrointestinal tract of the host, in order to reach the bacteria which, they ought to influence in the small intestines. The effects of these chemicals and digestive enzymes on MOS were investigated *in vitro* (Fig. 4).

The SCG derived MOS showed resistance to decomposition by these gastrointestinal products. The results agreed with the findings by Asano *et al.*,^23^ who showed that thermally produced MOS were resistant to human salivary amylase, artificial gastric juice, porcine pancreatic enzymes and rat intestinal mucous protein. The ability of MOS to tolerate the simulated gastrointestinal conditions shows that they are likely indigestible *in vivo* and would reach the large intestine without losing their structural integrity and, hence, maintain their prebiotic bioactivity.

### 2.5. TGA analysis of MOS

**Fig. 5:**
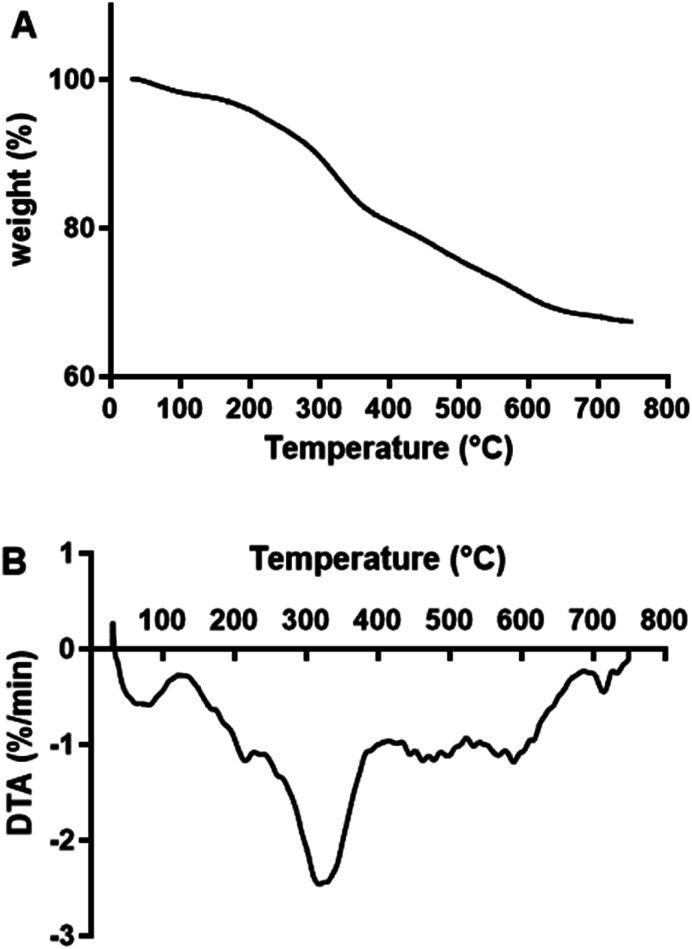
TGA (A) and DTA (B) curves of MOS.

TGA and DTA analysis were performed on freeze-dried MOS to establish their thermal stability (Fig. 5). MOS exhibited a slight loss in weight at approximately 50 °C due to moisture removal, followed by a more drastic weight loss between 150 and 400 °C. Similarly, López-Sanz *et al.*^24^ observed weight loss peaks for Vivinal galacto-oligosaccharides (GOS) at 36, 90 and 135 °C.

### 2.6. Prebiotic activity of MOS

**Fig. 6:**
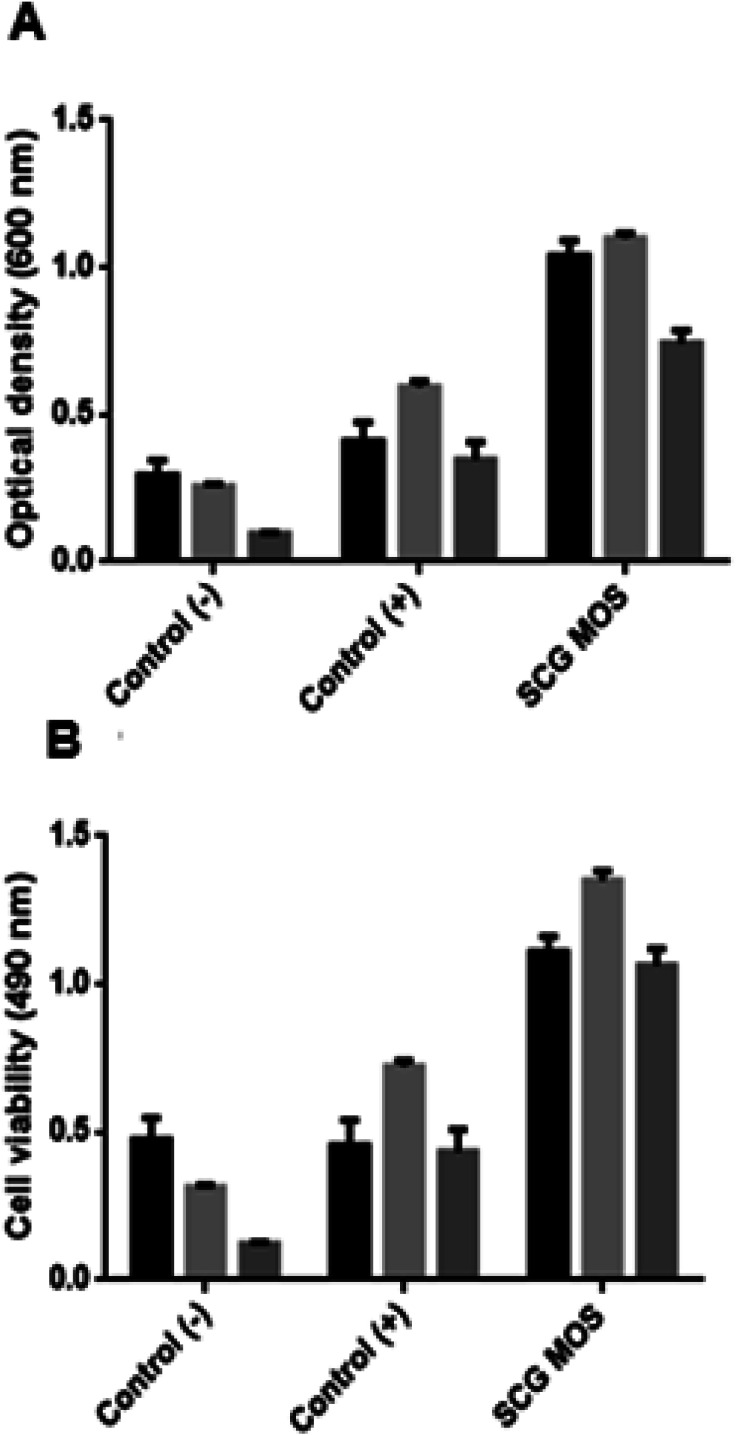
Effect of MOS on beneficial bacteria. Columns: black – *L. bulgaricus*, light grey – *B. subtilis* and dark grey – *S. thermophilus*. The (A) cell biomass growth (optical density) and (B) viability of beneficial bacteria in the presence and absence of various sugars as carbon sources. Control (−): sugar-free, control (+): glucose present and SCG MOS: MOS obtained from SCG.

The MOS were further tested for their effect on the growth of beneficial bacteria (Fig. 6). The MOS promoted the growth of *L. bulgaricus*, *S. thermophilus* and *B. subtilis*. And the viability of these beneficial bacteria after incubation with MOS correlates with the optical density, which means that the cells did not die upon MOS consumption.

Cao *et al.*^25^ and Srivastava *et al.*^26^ demonstrated similar findings when testing the effect of commercial MOS on *L. plantarum* and low DP MOS (M2 and M3) on seven *Lactobacillus* sp. Pan *et al.*^27^ also reported that MOS enhance the growth of *Lactobacilli* and *Bifidobacteria*, while inhibiting *Enterococcus* and *Enterobacteriaceae*. Magengelele *et al.*^9^ also reported that MOS derived from ivory nut mannan (INM), locust bean gum (LBG) and guar gum (GG) enhance the growth of *B. subtilis* and *S. thermophilus*. Numerous studies have reported that MOS inhibit the proliferation of pathogenic bacteria in the digestive tract of mammals in many ways which include the production of short-chain fatty acids (SCFAs) by beneficial bacteria, which contributes to the decrease in pH and inhibition of pathogens from attaching to the intestinal walls.^28,29^

### 2.7. Detection of SCFA production as a result of MOS fermentation

SCFAs are well-known for decreasing the pH in the digestive tract, resulting in the competitive exclusion of pathogens as well as triggering a host immune response.^30^ We proceeded to assess the fermentability of MOS to SCFAs by the beneficial bacteria; *B. subtilis*, *L. bulgaricus* and *S. thermophilus* (Table 2). *S. thermophilus* and *B. subtilis* failed to produce SCFAs in the negative control (carbon source free media). However, *L. bulgaricus* did produce SCFAs; this was expected as it is a lactic acid-producing bacterium.^27,28^ Audisio *et al.*^31^ and Baurhoo *et al.*^29^ reported that *Lactobacillus* sp. and *Bifidobacterium* could utilise glucose and produce lactic acid and acetic acid in the gut of birds. Production of these acids prevents pathogens from attaching to the host's intestinal walls.^28^

**Table 2:** Amount of putative SCFAs detected after MOS utilisation by beneficial bacteria. Values are represented as means ± standard deviations, *n* = 3

Carbon source	Putative SCFAs (mM)		
	*B. subtilis*	*S. thermophiles*	*L. bulgaricus*
Glucose	0.12 ± 0.03	0.06 ± 0.01	0.08 ± 0.01
No sugar	ND	ND	0.04 ± 0.01
SCG MOS	0.42 ± 0.03	0.34 ± 0.01	0.46 ± 0.02

All bacterial cells produced and secreted compounds bearing a carbonyl functional group, which we attributed to be the presence of SCFAs, to the media in the presence of the MOS. SCFAs, such as butyric acid, have been reported to inhibit the activity of pathogens like *Salmonella* and *Clostridium perfringens* in mammals. Wu *et al.*^30^ reported that supplementation of mice drinking water with acetate, butyrate and propionate reduces their susceptibility to *Klebsiella pneumoniae* infections. Our results are also in agreement with the findings of Pan *et al.*,^32^ who reported that MOS intake by *Bifidobacteria* and *Lactobacilli* improves the amount of SCFAs (butyrate, propionate and acetate) produced. The presence of SCFAs in the gut leads to a decrease in free fatty acids, reducing the risks of getting type-2 diabetes.^33^ Diabetes mellitus is one of the top ten leading causes of death globally. The ability of *L. bulgaricus*, *B. subtilis* and *S. thermophilus* to produce SCFAs after MOS utilisation will increase insulin sensitivity by reducing free fatty acids and ghrelin (the hunger hormone), reducing the risk of developing type-2 diabetes.^33^ Further studies are required to determine which type of SCFAs were produced by which bacteria and to quantify each SCFA, because these beneficial bacteria could be producing different SCFAs, which may have different functions in the gut.

### 2.8. Bacterial biofilm formation and auto-aggregation in the presence of MOS

The effect of MOS obtained from SCG on the formation of biofilms and auto-aggregation of beneficial bacteria was investigated. The results in Table 3 show biofilm growth normalised with total cell growth (OD_540_/OD_600_), and auto-aggregation percentage (%) for each bacterium. In terms of auto-aggregation influence, *L. bulgaricus*, *B. subtilis* and *S. thermophilus* grown in SCG derived MOS showed aggregation percentages of 18.21, 20.98 and 17.99%, respectively, and were better than cultures grown in the absence of a carbon source. Cao *et al.*^25^ reported that MOS enhanced the auto-aggregation of *L. plantarum* with a rate of auto-aggregation of 23.76%, which is slightly higher than that of *L. bulgaricus*, *B. subtilis* and *S. thermophilus* reported in the current study. The variations in the rate of auto-aggregation could be due to the use of different beneficial bacterial species. The DP of the commercial MOS used by Cao *et al.*^25^ may also be different from the MOS used in this study. These three probiotic bacteria were also able to form biofilms in the presence of MOS. It has been reported that the ability of beneficial bacteria to attach to surfaces, *i.e.*, epithelial cells, provides them with an opportunity to block pathogens from also attaching to these surfaces.^34^ Magengelele *et al.*^9^ also showed that MOS derived from INM, LBG and GG improved biofilm formation in *S. thermophilus* and *B. subtilis*. There is no literature available on the effect of SCG-derived MOS on auto-aggregation and biofilm formation of bacteria. However, Cao *et al.*^25^ reported that commercial MOS enhanced auto-aggregation of *L. plantarum* ATCC14917. MOS also had a positive effect on biofilm formation of *L. plantarum* ATCC14917 on mucin (24.65%) and Caco-2 cells (14.71%).^25^ SCG-derived MOS enhanced the ability of *S. thermophilus*, *B. subtilis* and *L. bulgaricus* to form microcolonies and produce biofilms.

**Table 3:** The effect of *Bacillus* sp.-derived mannanase produced MOS (2% (w/v) reducing sugar basis) on the auto-aggregation and biofilm formation of beneficial bacteria. Values are represented as means ± standard deviations, *n* = 3

Carbon source	*L. bulgaricus*	*B. subtilis*	*S. thermophilus*
**Biofilm formation (OD**_**540**_ **/OD**_**600**_ **)**			
No sugar	0.79 ± 0.01	0.63 ± 0.03	0.62 ± 0.02
Glucose	0.94 ± 0.40	0.55 ± 0.06	0.66 ± 0.01
SCG MOS	2.19 ± 0.44	1.28 ± 0.14	1.19 ± 0.11

**Auto-aggregation (%)**			
No sugar	1.63 ± 0.13	1.79 ± 0.43	4.19 ± 0.34
Glucose	3.06 ± 0.19	2.72 ± 0.23	4.70 ± 0.24
SCG MOS	18.21 ± 1.35	20.98 ± 2.21	17.99 ± 2.73

## 3 Experimental

### 3.1. Pretreatment of SCG

SCG was supplied by National Brands Limited (Island, South Africa). For the pretreatment of SCG, 12.5 g of NaOH, 1 L of distilled water and 50 g of SCG were added in a 1 L Schott bottle. The slurry was incubated at 70 °C with occasional stirring for 4 hours in a 6 Litre water bath (Labnet International. Inc, Woodbridge, USA). After incubation, the slurry was cooled down to room temperature, filtered using a cheesecloth and washed three times with 500 mL of distilled water. The SCG was then dried at 50 °C until a constant weight was obtained. Dried NaOH treated SCG was pulverised using a pestle and mortar, passed through a sieve (mesh size 0.5 mm) and kept at room temperature until use. Untreated SCG (50 g) was also washed, dried and pulverised as the pretreated SCG.

### 3.2. Structural analysis of SCG

#### 3.2.1. Fourier transform infrared spectrometer (FT-IR) analysis

A Spectrum 100 FT-IR spectrometer system (PerkinElmer, Welles-ley, MA) was used to characterise the NaOH pretreated and untreated SCG. A spring-loaded anvil was used to evenly press the samples against the spotting surface. FT-IR spectra were obtained by averaging sixty-four scans from 4000 to 600 cm^−1^. Spectrum™ One software was used for baseline and ATR corrections for penetration depth and frequency.

#### 3.2.2. Thermogravimetric (TGA) analysis

TGA was performed using a PerkinElmer analyser (PerkinElmer, Shelton, CT, USA). For analysis (pretreated and untreated SCG), about 3 mg of the sample was placed in a platinum pan. A stream of nitrogen at 20 mL min^−1^ was run over the incubation chamber during analysis. The sample was heated between 30 °C and 750 °C, with an increase of 30 °C min^−1^ under nitrogen gas.

### 3.3. Enzymatic generation of MOS from SCG

Prior to hydrolysis, the pretreated SCG was pre-wetted in 50 mM phosphate buffer (pH 7.0) for 5 hours. After this, 4% (w/v) or 40 mg mL^−1^ SCG was hydrolysed by 0.25 mg protein per g SCG of *Bacillus* sp. derived mannanase, Man26A (Megazyme, Bray, Ireland). The reaction mixture also contained 1 mg mL^−1^ of bovine serum albumin to minimise non-productive binding of the enzyme to the non-carbohydrate fraction of SCG. The reaction mixtures were incubated at 50 °C for 48 hours, with agitation at 70 rpm. After this, the reaction was stopped by incubating the samples at 100 °C for 5 minutes. The samples were then centrifuged at 16 060 × *g* for 5 minutes using a Biofuge pico Heraeus centrifuge (Hanau, Germany) and the supernatant subsequently quantified for reducing sugar release using the DNS method as described in literature.^35^

### 3.4. Analysis of SCG hydrolysis products by high-performance liquid chromatography (HPLC)

The MOS were identified and quantified using a CarboSep CHO 411 column (Concise Separations, San Jose, USA) connected to a Shimadzu HPLC system with a refractive index detector (Shimadzu Corp, Kyoto, Japan) as described in our previous work.^9^

### 3.5. Gastrointestinal tolerance test of MOS

Bile salts (0.3% w/v), α-amylase (1 mg mL^−1^) and trypsin (1 mg mL^−1^) (Sigma-Aldrich, St. Louis, USA) were prepared in 50 mM phosphate buffer (pH 7.0). The prepared solutions of bile salts, α-amylase and trypsin were mixed with MOS derived from SCG (1.7 mg mL^−1^ reducing sugar basis), in a 1 : 1 ratio. A 50 mM phosphate buffer (pH 7.0) was used as the negative control. For hydrochloric acid, the phosphate buffer that was used to prepare SCG MOS was removed using a Centrivap vacuum concentrator system (Labconco, Boston, New York), and 1 mL of 0.1 M HCl (pH 1.5) was used to resuspend the MOS. The solutions were all incubated at 37 °C for 4 h. After incubation, the Eppendorf tubes with α-amylase and trypsin were incubated at 100 °C for 5 minutes to inactivate the enzymes, and the HCl was removed using the Centrivap vacuum concentrator system and replaced with 1 mL of phosphate buffer. The reducing sugars were quantified using the DNS method described before. A known amount of mannose (1.7 mg mL^−1^ reducing sugar basis) was used in under the assayed conditions to correct for sugar losses.

### 3.6. TGA analysis of SCG MOS

TGA was performed on the produced MOS, after lyophilization, using a PerkinElmer analyser as described in Section 3.2.2.

### 3.7. Prebiotic activity determination of MOS

Bacterial cells (1 mL) stored in Luria broth were harvested by centrifugation at 16 060 × *g* using a Biofuge pico Heraeus (Hanau, Germany) desktop centrifuge for 5 minutes and resuspended in saline (0.9% (w/v) NaCl) before measuring their optical density at 600 nm. The *in vitro* fermentation of probiotics was performed in 1× M9 minimal media (3.4% (w/v) K_2_HPO_4_; 1.5% (w/v) KH_2_PO_4_; 0.5% (w/v) NH_4_Cl; 0.25% (w/v) NaCl) with a pH of 7.4. The samples were supplemented with MOS at a final concentration of 0.2% ((w/v), reducing sugar content). The initial absorbance (600 nm) of the reaction mixture was adjusted to 0.1 for all the samples. This was followed by incubation of the samples at 37 °C for 7 hours, with shaking at 150 rpm. The positive control was supplemented with 0.4% (w/v) glucose, while the negative control was sugar-free. The optical density of the samples was measured. The viability of the cells was subsequently tested by adding 50 μL of 0.02% (w/v) of iodonitrotetrazolium chloride into a 96-well plate containing 200 μL of the cells that were grown for 7 hours with the different carbon sources (no carbon source or glucose and/or MOS). The plate was incubated for 1 hour and the absorbance measured at 490 nm.

### 3.8. Influence of MOS on short chain fatty acids (SCFAs) synthesis by bacteria

After the prebiotic study, a liquid–liquid extraction was performed to extract SCFAs from the culture broths using diethyl-ether and NaOH as described previously.^36^ The SCFAs were quantified by absorbance reading at 210 nm with butyric acid used as a suitable standard.

### 3.9. Influence of MOS on auto-aggregation of bacteria

The influence of MOS on the auto-aggregation nature of bacteria was studied as described previously.^28^ Bacterial cells were harvested by centrifugation at 16 060 × *g* for 5 minutes and the pellet was resuspended in 1× M9 minimal media. The absorbance of the bacteria was adjusted to an optical density reading of 0.5 ± 0.05 in test tubes using 0.4% (w/v) glucose and 0.2% (w/w) MOS derived from SCG hydrolysis. Then, the bacterial suspensions were incubated at room temperature, the absorbance readings were measured at *t* = 0 and *t* = 1 h. Auto-aggregation percentage is expressed as follows:Autoaggregation = [1 − *A*_*t*_/*A*_0_] × 100where, *A*_*t*_ represents the absorbance at time *t* = 1 h and *A*_0_ is the absorbance at *t* = 0.

### 3.10. Biofilm formation influence of MOS on bacteria

The ability of MOS to influence biofilm formation was monitored by using sterile 96-well flat-bottom microtiter plates. Briefly, the plates were prepared for each strain with four replicates of 1× M9 minimal media supplemented with different 0.2% (w/v) sugars (buffer alone, glucose, SCG MOS) (final bacterial OD read of 0.5). A plate for each strain was covered and incubated without shaking at 37 °C for 24 h. After the incubation period, the wells in each plate were washed three times with phosphate-buffered saline (PBS) solution to remove unbound cells. The cells attached to the wall of each well were stained for 20 min with 250 μL of 0.1% (w/v) crystal violet in water and washed again three times with the PBS solution to remove unbound crystal violet. Bound cells were quantified by adding 250 μL of acetone/ethanol (20 : 80 (v/v)), followed by measuring absorbance at 540 nm. For comparison of biofilm formation among strains in each medium, the total growth was monitored first by measuring optical density in each well, and then normalizing the biofilm growth with its total cell growth values.

## 4 Conclusions

MOS were successfully produced from NaOH pretreated SCG using a *Bacillus* sp. derived *endo*-1,4-β-mannanase, with M2 and M3 as the predominant products. TGA and simulated gastrointestinal tolerance analysis showed that the MOS exhibit good properties for application as functional foods. Finally, the MOS exhibited the ability to enhance the growth of beneficial bacteria (*L. bulgaricus*, *B. subtilis* and *S. thermophilus*), which, in turn, produced SCFAs. During this, the MOS improved the ability of the beneficial bacteria to adhere to surfaces, which could be useful measure in offering protection against environmental stress.

## Author contributions

Mihle Magengelele: methodology, formal analysis, investigation, visualization, writing – original draft. Samkelo Malgas: conceptualization, formal analysis, investigation, methodology, project administration, supervision, validation, writing – review & editing. Brett I. Pletschke: conceptualization, formal analysis, funding acquisition, methodology, project administration, resources, supervision, validation, writing – review & editing.

## Conflicts of interest

There are no conflicts to declare.

## Supplementary Material
